# Sequence Evidence in the Archaeal Genomes that tRNAs Emerged Through the Combination of Ancestral Genes as 5′ and 3′ tRNA Halves

**DOI:** 10.1371/journal.pone.0001622

**Published:** 2008-02-20

**Authors:** Kosuke Fujishima, Junichi Sugahara, Masaru Tomita, Akio Kanai

**Affiliations:** 1 Institute for Advanced Biosciences, Keio University, Tsuruoka, Yamagata, Japan; 2 Systems Biology Program, Graduate School of Media and Governance, Keio University, Fujisawa, Japan; 3 Department of Environment and Information, Keio University, Fujisawa, Japan; Tata Institute of Fundamental Research, India

## Abstract

The discovery of separate 5′ and 3′ halves of transfer RNA (tRNA) molecules—so-called split tRNA—in the archaeal parasite *Nanoarchaeum equitans* made us wonder whether ancestral tRNA was encoded on 1 or 2 genes. We performed a comprehensive phylogenetic analysis of tRNAs in 45 archaeal species to explore the relationship between the three types of tRNAs (nonintronic, intronic and split). We classified 1953 mature tRNA sequences into 22 clusters. All split tRNAs have shown phylogenetic relationships with other tRNAs possessing the same anticodon. We also mimicked split tRNA by artificially separating the tRNA sequences of 7 primitive archaeal species at the anticodon and analyzed the sequence similarity and diversity of the 5′ and 3′ tRNA halves. Network analysis revealed specific characteristics of and topological differences between the 5′ and 3′ tRNA halves: the 5′ half sequences were categorized into 6 distinct groups with a sequence similarity of >80%, while the 3′ half sequences were categorized into 9 groups with a higher sequence similarity of >88%, suggesting different evolutionary backgrounds of the 2 halves. Furthermore, the combinations of 5′ and 3′ halves corresponded with the variation of amino acids in the codon table. We found not only universally conserved combinations of 5′–3′ tRNA halves in tRNA^iMet^, tRNA^Thr^, tRNA^Ile^, tRNA^Gly^, tRNA^Gln^, tRNA^Glu^, tRNA^Asp^, tRNA^Lys^, tRNA^Arg^ and tRNA^Leu^ but also phylum-specific combinations in tRNA^Pro^, tRNA^Ala^, and tRNA^Trp^. Our results support the idea that tRNA emerged through the combination of separate genes and explain the sequence diversity that arose during archaeal tRNA evolution.

## Introduction

The origin and evolution of tRNA is an exciting topic widely discussed in the field of molecular evolution [1]–[5]. Three types of tRNA genes have been identified in archaeal genomes: “nonintronic tRNA”, which is encoded on a single gene with no intron sequence; “intron-containing tRNA” (intronic tRNA), which is encoded on a single gene with 1 to 3 introns punctuating the mature tRNA sequence at various locations [6]–[8]; and “split tRNA”, which has been found only in the hyperthermophilic archaeal parasite *Nanoarchaeum equitans*, of which the 5′ and 3′ halves are encoded on separate genes [9]. The discovery of these split tRNA genes raised the question of whether ancestral tRNA was encoded on a single gene or separate genes. No redundant tRNAs with the same anticodon have been found in the *N. equitans* genome, and experimental data suggest that these split tRNAs are transcribed and processed into functional tRNA molecules [9]. The joiner region of the pre-tRNA product of the 2 split tRNA genes consists of a bulge–helix–loop (BHL) motif, a relaxed form of bulge–helix–bulge (BHB) motif which is recognized and cleaved by homodimer or heterotetramer splicing endonucleases during tRNA processing [10], [11]. Interestingly, BHB and BHL motif is known as a distinctive structure for protein-spliced intron and are observed at the exon–intron boundary of archaeal intronic tRNA [12], [13], mRNA [14], [15], rRNA [16] and eukaryal intronic tRNAs [17], [18]. Recently, a novel permuted structure of tRNA gene was discovered in a genome of primitive red alga, *Cyanidioschyzon merolae*, which tRNAs are encoded on a single gene in the order of 3′ half–5′ half forming a potential BHB motif around intron-exon junction [19]. These common processing machineries suggest an evolutionary link among various types of tRNAs.

In this context, Di Giulio suggested that ancestral tRNA was encoded by 2 separate minigenes, which later fused to encode modern tRNAs [3], [5], [20]. This conclusion is based on the probabilistic argument that the boundaries of the split tRNAs and of many of the introns found in the tRNA sequences are located at anticodon loops. In addition, it has been proposed that the tRNA molecule originated by direct duplication of an RNA hairpin structure [3], [4], [21]. Experimental studies have shown that a single hairpin minihelix that recapitulates the domain of top half tRNA sequence (acceptor stem+TψC arm helix) can be aminoacylated by modern tRNA synthetases [22]. The definition of the minihelix is different from that of split tRNA (separated at the anticodon loop) but the idea of tRNA emerged from two distinct minigenes has been supported by the genomic tag hypothesis, which proposes that the top half tRNA sequence must have originated among ancient life to catalyze RNA replication in the ‘RNA world’ ahead of bottom half tRNA sequence (D arm helix+anticodon stem) on account of the independent structure and functionality of the 5′ and 3′ tRNA sequences [1], [2]. Nevertheless, the phylogenetic position of *N. equitans* remains uncertain, as it has two incongruent positions in the phylogenetic tree of the Archaea depending on the dataset [23], [24], and so the question remains as to whether the split tRNA genes really exemplify the ancestral form of tRNA.

We have recently developed software called SPLITS [7], [8], which has predicted most of the archaeal missing tRNAs that cannot be predicted by tRNAscan-SE, the most widely used software for tRNA annotation [25]. The recent accumulation of complete archaeal genome sequences in the public databases enabled us to perform comprehensive phylogenetic analysis of 1953 archaeal tRNA sequences in 45 archaeal species. Here, we report on the overall phylogeny of the archaeal tRNAs, focusing on the evolutionary relationships among nonintronic, intronic, and split tRNAs. Further, by separating all kinds of tRNAs at the anticodon region and mimicking the split tRNA sequences, we show that network topologies based on the sequence similarities of 5′ and 3′ tRNA halves differ significantly. Moreover, the specific combination of 5′ and 3′ tRNA halves from different groups explained the variation of amino acids in the codon table. Here, we provide sequence evidence supporting the hypothesis that tRNA evolved through a combination of 5′ and 3′ tRNA sequences.

## Results and Discussion

### Phylogenetic analysis of mature tRNAs in 45 archaeal species

**Figure 1:**
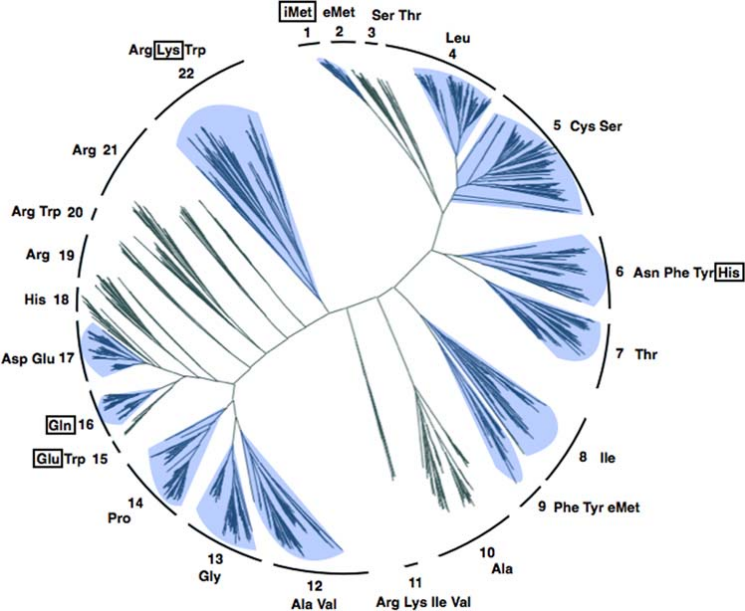
Phylogenetic tree of mature tRNA sequences in 45 archaeal species. The phylogenetic neighbor-joining tree was constructed by using mature sequences of 1953 predicted tRNAs from the complete genomes of 45 archaeal species. Clusters are numbered from 1 to 22, and tRNAs within each cluster are denoted by amino acids corresponding to the anticodon. Clusters including tRNAs from all 3 archaeal phyla (Euryarchaeota, Crenarchaeota, and Nanoarchaeota) are shaded in blue. The amino acids corresponding to the split tRNAs are boxed.

We predicted 1977 putative tRNA candidates from the genome sequences of 45 archaeal species with 2 tRNA predicting programs, SPLITS [7], [8] and tRNAscan-SE [25]. All tRNA sequences were manually checked, and 24 false candidates (tRNA-like sequences used for viral integration, and pseudogenes) were eliminated from the dataset. The resulting 1953 archaeal tRNAs, including 6 known split tRNAs and 423 intronic tRNAs, were used as a dataset for phylogenetic analysis. We performed structural alignment based on their mature tRNA sequences (from which introns and leader sequences were deleted) by manually improving the multiple alignment data through complete matching of the consensus nucleotides conserved among archaeal tRNAs (see Methods for detail). An unrooted neighbor-joining (NJ) tree was then produced. As a result, 1953 tRNAs were separated into 22 clusters: 12 dominated by tRNAs with its anticodon corresponding to a single type of amino acid (e.g., a tRNA for Ala), and 10 consisting of tRNAs with anticodons corresponding to 2 to 4 amino acids (e.g., a tRNA for Arg-Lys-Trp [i.e., 3 amino acids]) (Fig. 1). For example, 89 out of 90 archaeal tRNA^Gln^ were clustered in the same branch (cluster 16); and all tRNA^Asp^ and tRNA^Glu^ except the split tRNA^Glu^ were clustered indistinguishably in the same branch (cluster 17). Like as Asp-Glu cluster, there are several indistinguishable pairs of amino acids clustered in the same branch that are in precursor-product relationship or in biosynthetic relationship (ex: Ala-Val, Arg-Lys, Phe-Tyr, Cys-Ser) supporting the coevolution theory of the origin of the genetic code [26].

The split tRNAs were placed in 5 different clusters: 4 split tRNAs (tRNA^iMet^ [cluster 1], tRNA^His^ [cluster 6], tRNA^Gln^ [cluster 16], and tRNA^Lys^ [cluster 22]) were placed adjacent to other tRNAs with synonymous codons. Only the two split tRNA^Glu^ (cluster 15) was distant from any other tRNAs that branched at the root of the tRNA^Gln^ cluster. Accordingly, only 13 tRNA clusters (shaded in Fig. 1) included tRNAs from all 3 archaeal phyla (Nanoarchaeota, Crenarchaeota, and Euryarchaeota), suggesting that about half of the tRNAs corresponding to specific amino acids evolved from a monophyletic origin. For a clearer understanding of the phylogenetic distribution of archaeal tRNAs, we tabulated the phylogenetic location of tRNAs from 16 archaeal genera against each tRNA's cluster number (Supplemental Fig. S1). Further, we have found that none of the *N. equitans* tRNAs branched adjacent to the tRNAs of the host species *Ignicoccus hospitalis* (data not shown), thus we assume that no tRNA genes were laterally transferred between the 2 species after *N. equitans* became parasite of *Ignicoccus* cell.

**Figure 2:**
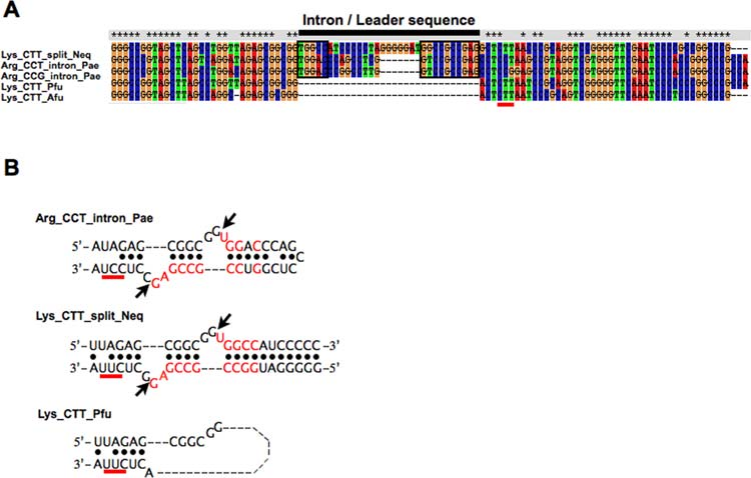
Comparison of the 3 types of tRNA sequences. (A) Full nucleotide sequences of pre-tRNAs (1 split tRNA^Lys^ [Neq] and 2 intronic tRNA^Arg^s [Pae]) and 2 nonintronic tRNA^Lys^ (Pfu and Afu) were aligned. Black bar marks the intron of the intronic tRNAs and the leader sequences of the split tRNAs, which are inserted at tRNA nucleotide position 32/33. Red bar marks the anticodon. (B) Comparison of the secondary structures and nucleotide sequences around the exon–intron boundary of the 3 types of tRNAs. Nucleotides that are identical between leader sequence and intron are shown in red. Red bar marks the anticodon.

Since 4 out of the 6 split tRNAs possessed clear sequence similarity with other tRNAs with the same anticodons, we focused on the precise phylogeny of the 6 split tRNAs from *N. equitans* in relation to other archaeal tRNAs. We performed detailed phylogenetic analysis for each of the 6 split tRNAs based on the Bayesian method (Supplemental Fig. S2). Mature sequences of 3 split tRNAs (tRNA^iMet^, tRNA^Lys^ and tRNA^Gln^) branched with other tRNAs with same anticodon from Crenarchaeota and Euryarchaeota lineages. Split tRNA^His^ clustered with tRNA^His^ from the Crenarchaeota lineage and *M. kandleri*. The most notable was split tRNA^Glu^, which located at the root of tRNA^Glu^ cluster. The phylogenetic position of split tRNA^Glu^ in NJ tree was adjacent to tRNA^Gln^ and tRNA^Trp^ cluster, although this contradiction exemplifies the sequence ambiguity of split tRNA^Glu^. Thus, split tRNAs reveal various characteristics in the phylogeny of archaeal tRNAs—universal (tRNA^iMet^, tRNA^Lys^ and tRNA^Gln^), crenarchaeal-specific (tRNA^His^), and unique (tRNA^Glu^) phylogenetic positions—suggesting that split tRNA could be the ancestral form of tRNAs. Besides, intronic tRNAs were scattered throughout tRNA phylogeny in almost every tRNA clusters with introns positioned at the same location as the 5′–3′ boundary of the split tRNAs. We found an intronic tRNA^Arg^ with an intron sequence possessing 58% identity to that of the split tRNA^Lys^ leader sequence located at the same position as that of the intron position (Fig. 2). Both tRNA belongs to the same cluster (cluster 22 in Fig. 1) suggesting that some intronic tRNAs may have emerged from integrated split tRNA in the archaeal genome.

### Sequence analysis of the 5′ and 3′ tRNA halves reveals their different evolutionary backgrounds

**Figure 3:**
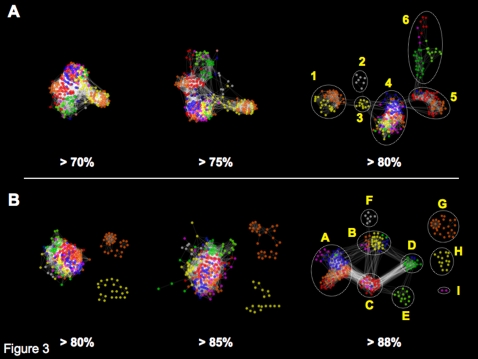
Network analysis based on the sequence similarities of 5′ and 3′ tRNA halves. A total of 296 mature tRNA sequences from 7 archaeal species (Neq, Sso, Ape, Pae, Mka, Pfu, Mja) were artificially split into 5′ and 3′ halves at the anti-codon region. Each node (colored dot) represents a tRNA half, and its color indicates the charged amino acid's chemical properties (DE, mid-green; MNQ, light green; RKH, blue; FWY, purple; AGP, red; ILV, orange; CST, yellow; iMet, gray). Nodes are linked by a white line (edge) when the sequence similarity is above the threshold. (A) Network created by set of 5′ half sequences with thresholds of >70%, >75%, and >80%. The sequences are classified into 6 clusters (1–6) at a threshold of >80%. (B) Network created by set of 3′ half sequences with thresholds of >80%, >85%, and >88%. The sequences are classified into 9 clusters (A–I) at a threshold of >88%.

As described in the previous section, comprehensive phylogenetic analysis of 1953 mature archaeal tRNA sequences revealed an evolutionary relationship between the three types of tRNAs. If split genes were the ancestral form of tRNA, can we explain the origin and evolution of modern tRNA sequences by the combination patterns of 5′ and 3′ tRNA halves? We attempted to corroborate this hypothesis by analyzing the evolutionary backgrounds of 5′ and 3′ halves. We selected 7 archaeal species, each from the closest taxon to the last archaeal common ancestor: 1 Nanoarchaeota (*N. equitans*, Neq), 3 Crenarchaeota (*P. aerophilum*, Pae; *A. pernix*, Ape; and *S. solfataricus*; Sso), and 3 Euryarchaeota (*P. furiosus*, Pfu; *M. kandleri*, Mka; and *M. jannaschii*, Mja). In total, 296 tRNA sequences were extracted and separated into the 5′ half (positions 1–33) and the 3′ half (positions 37–73) at the anticodon region (positions 34–36) to mimic split tRNA. We considered each tRNA half as a network node, and nodes were linked when sequence identity between the 2 tRNA halves was above certain threshold. Accordingly, we have constructed 2 different networks (5′ half network and 3′ half network) based on their sequence similarities. Previously, a network-based approach was used to explain 2 mechanisms of tRNA evolution: point mutation and complementary mutation [27]. Thus, concept of representing the sequence similarities among tRNA halves as a network provides comprehensive understanding of sequence characteristics and its diversity based on the global statistics. Figure. 3 shows the sequence similarity networks of 5′ and 3′ tRNA halves with thresholds of >70%, >75%, and >80% for 5′ tRNA halves (Fig. 3A), and >80%, >85%, and >88% for 3′ tRNA halves (Fig. 3B). A noticeable topological difference is apparent between the sequence similarity networks of the 5′ and 3′ halves. Sequence diversity of the 5′ half sequences appeared between sequence similarities of >70% and >75%, where the single large network started to localize into small clusters corresponding to specific amino acids. This feature was more prominent at a similarity of >80%, where 5′ halves clearly separated into 6 groups (1–6 in Fig. 3A). In contrast, all 3′ half sequences except tRNA^Ser^ and tRNA^Leu^ (possessing long variable stem loops shown as yellow and orange nodes in Fig. 3B) gathered into 1 large group even at a sequence similarity of >80%. The 3′ half sequences were finally differentiated at a sequence similarity of >88% into 9 groups (A–I in Fig. 3B).

**Figure 4:**
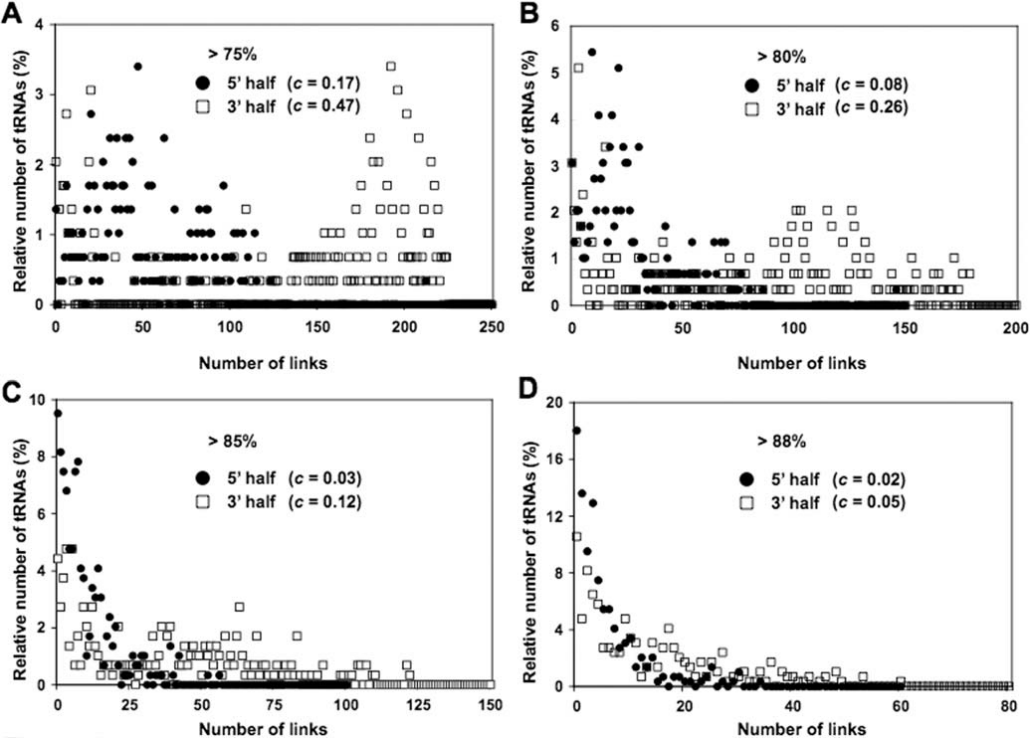
Distribution of networks based on the 5′ and 3′ tRNA sequences. Relation between the number of tRNA sequences and the number of links is represented at 4 sequence similarity thresholds (A–D). Clustering coefficient *c* is denoted for each tRNA halves.

To characterize the overall network topology of the 5′ and 3′ halves, we compared the 2 networks by measuring the distribution of the number of connections per node (connectivity distribution). Network topology can be classified into 2 distinct types: ‘scale-free networks’, in which a few nodes have many links but most have only a few links, and which follow a power-law distribution [28]; and ‘small-world networks’, which consist of nodes linked randomly and follow a Poisson or exponential distribution [29]. We found obvious difference in degree distribution between the 2 networks at all similarity thresholds (Fig. 4). At a similarity threshold of >75%, the clustering coefficient *c* (degree of linkage between nodes) of 5′ half was only 0.17 while 3′ half was 0.47, which means that 47% of all possible connection are used. The power-law-like distribution of the 5′ half network was enhanced as the similarity threshold increased, while the distribution of 3′ half network changed from Poisson-like (>75%) to irregular (>80–85%) then finally a power-law-like distribution at a sequence similarity of >88%. Meanwhile, the ratio of the cluster coefficient between 5′ and 3′ halves were constantly 1∶2.5–1∶4 showing that characteristics of the 2 tRNA domains significantly differ at all level of sequence threshold (75%–88%). We suggest that high commonality of the 3′ half sequences is one of the evidence of which 3′ half could have had a single origin. On the other hand, the scale-free distribution of the 5′ half represents a specific sequence characteristic within each sequence group, suggesting a non-monophyletic origin of the 5′ half sequences. These different sequence characteristics of the 5′ and 3′ tRNA halves further support the idea that ancient archaeal tRNA emerged from a specific combination of 5′ and 3′ halves.

### Specific combinations of 5′ with 3′ tRNA halves strongly correlate with amino acid distribution in the codon table

**Figure 5:**
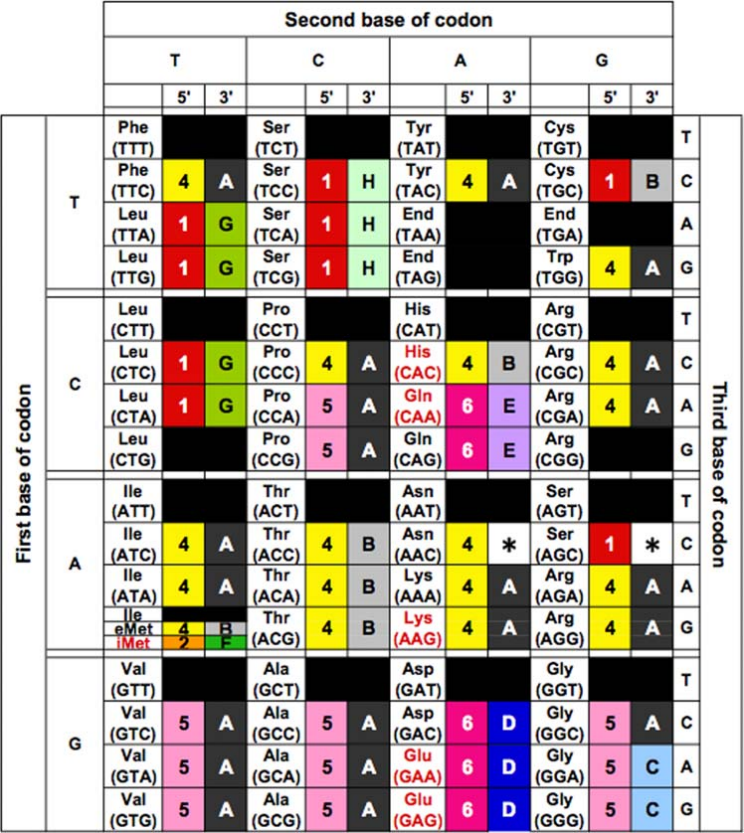
Representation of *N. equitans* codon table filled with 5′ and 3′ tRNA halves. The table is filled with the group IDs (see Fig. 3) corresponding to each of the 5′ and 3′ tRNA halves in *N. equitans* (Neq). The anti-codon corresponding to the 6 split tRNAs is shown in red. An asterisk indicates that a sequence does not have a similar sequence above the threshold (5′ half, 80%; 3′ half, 88%).

**Figure 6:**
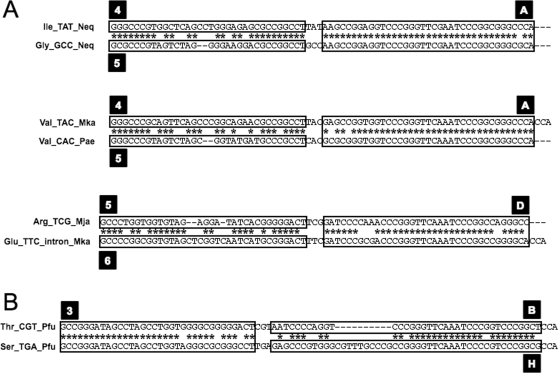
Examples of tRNA sequences explained by specific combinations of 5′ and 3′ tRNA halves. (A) Examples of tRNA sequences with different 5′ half sequences but a common 3′ half sequence. (B) Example of tRNA sequences with different 3′ half sequences but a common 5′ half sequence. Asterisk denotes matching nucleotides in the aligned tRNA sequences. Boxes delineate sequences belonging to the same group in the sequence similarity network (Fig. 3). The corresponding group ID is labeled in a black box.

Since the 5′ and 3′ halves are suggested to have had different evolutionary backgrounds, we hypothesized that the sequence variety of tRNAs was derived directly from different combinations of 5′ and 3′ tRNA sequences in the early stage of archaeal evolution. To examine the correlation between specific combinations of 5′ and 3′ halves and the corresponding amino acids in the codon table, we filled the codon table of 7 archaeal species by annotating each of the anticodons with the 5′ and 3′ group IDs denoted in Figure. 3. We have selected different sequence similarity thresholds (80% for 5′ half and 88% for 3′ half) for grouping the tRNA sequences, since the two networks possessed similar clustering coefficient of *c* = 0.08 and 0.05. Figure. 5 shows a codon table filled by the 5′ and 3′ group IDs of *N. equitans* tRNA sequences. Codon tables of the other 6 species are shown in Supplemental Figure S3. Group A is the largest group in the 3′ half network, accounting for 104/296 (35%) sequences of tRNAs corresponding to various amino acids. In *N. equitans*, 3′ half sequences of tRNA^Arg^, tRNA^Ile^, tRNA^Val^, tRNA^Ala^, tRNA^Pro^, tRNA^Trp^, and tRNA^Phe^ were all categorized as group A. Adding the 5′ sequence information further classified these tRNAs into small groups possessing the same 5′–3′ combination. For example, in all 7 species, the same 4–A combination was observed for tRNA^Arg^ and tRNA^Lys^; this, together with the fact that split tRNA^Lys^ is clustered among the Arg-Lys cluster (Fig. 1), suggests that these tRNAs originated from a combination of ancestral minigenes of the 2 sequence groups. The same features are apparent in the 5–A combination of tRNA^Val^. Exceptionally, however, 2 tRNA^Val^s in *M. kandleri* both have the 4–A combination located in unique cluster (cluster 11 in Fig. 1). *M. kandleri* is an intriguing species which possesses many unique tRNAs clustered at different phylogenetic positions from other tRNAs with the same anticodon (Supplemental Fig. 1). The *N. equitans* genome also encodes unique tRNA^Ile^, which displays the only UAU (TAT) anticodon found in the Archaea. tRNA^Ile^ (Neq) is considered to be evolutionarily unique, as no homologous sequence has been found in any other archaeal species and therefore it is located within an isolated branch with various tRNAs from *M. kandleri* (cluster 11 in Fig. 1). However, by splitting the sequence of tRNA^Ile^ (Neq), we found tRNA^Gly^(GCC) in *N. equitans* genome which possessed identical 3′ half but different 5′half (Fig. 6A). This sequence evidence supports our idea of the ancient integration of split tRNA genes.

Comparing the codon tables of all 3 archaeal species groups (Supplemental Fig. S3), we found several interesting features related to the evolutionary selection of 5′–3′ combinations. We identified a phylum-specific distribution of tRNA halves corresponding to the same amino acids. For example, the 5′ tRNA sequences of euryarchaeal tRNA^Pro^ belong to group 6, but those of the nanoarchaeal and crenarchaeal tRNA^Pro^s belong to group 5. In contrast, the 3′ sequences of tRNA^Pro^ all belong to group C except *N. equitans*. tRNA^Trp^ and tRNA^Ala^ also showed phylum-specific 5′ half sequences. This different ancestry of the 5′ half sequences clearly explains the non-monophyletic origins of tRNA^Pro^, tRNA^Trp^ and tRNA^Ala^. Secondly, specific 5′–3′ combinations of tRNA^iMet^, tRNA^Thr^, tRNA^Ile^, tRNA^Gly^, tRNA^Gln^, tRNA^Glu^, tRNA^Asp^, tRNA^Lys^, tRNA^Arg^ and tRNA^Leu^ are conserved among all 7 species, placing these tRNAs among the most ancient unchanged tRNAs in the genome since the last archaeal common ancestor. Finally we observed a clear rule in the sequence divergence of 5′ and 3′ halves. For example in 5′ half network, group 1 has connection with group 3 but not with group 5 nor group 6 and in 3′ half network, group D has connection with group C and group B but not with group A (Fig. 3). Based on these rules, we found that group 5 and group C were placed at the center of network diversity, suggesting that these cluster should have been the origin of the whole network. When conbining the two (5′ and 3′) rules together, tRNA^Gly^ appeared as the only 5–C combination conserved among 7 species. Thus we suggest that minigenes encoding 5′ and 3′ tRNA sequence of tRNA^Gly^ were the origins of other tRNA genes in the very early stage of tRNA evolution. Later, gene duplication and sequence divergence occurred and selection of specific 5′–3′ tRNA sequences would have increased the specificity of tRNAs along with the usage of various amino acids. As shown in Figure 6, many tRNAs share identical 3′ halves but have different 5′ halves (Fig. 6A–6C), or vice versa (Fig. 6D). In that circumstance, the anticodon sequence could have been supplied by either sequences, a feature observed in split tRNAs [30].

In conclusion, our hypothesis that modern tRNAs evolved through the combination of 5′ and 3′ tRNA fragments is supported strongly by the variation in amino acids in the codon table and explains the sequence diversity in archaeal tRNAs. Some tRNAs show a highly conserved 3′ half but different 5′ half sequences (or vice versa), while other tRNAs consist of specific 5′–3′ combinations and correspond to single types of amino acids, features that are conserved throughout all archaeal phyla. We need natural or experimental evidence of how these split genes evolved into modern tRNA genes. According to current studies of processed pseudogenes, at least some rRNAs, mRNAs, and tRNAs can retropose (reverse-transcribe and integrate) into the genome in Mammalia and plants [31]–[33]. A good example is known as short interspread nuclear element (SINE); a retrotransposon which is known to be originated from tRNA via retropositional mechanism [34], [35]. Experimentally, *Escherichia coli*–derived tRNA^Tyr^ was reverse-transcribed as mono-cDNA by using retroplasmid-derived reverse transcriptase without any template DNA [36]. Therefore, we speculate that ancestral 5′ and 3′ tRNA minigenes integrated at the transcript level, along with the help of reverse transcriptase or integrase. If so, both intronic and nonintronic tRNAs could have been produced from transcribed split tRNA, resulting in the integration of the leader sequence of split tRNA as an intron sequence containing the BHB motif, and the deposition of the CCA sequence added by nucleotidyltransferase downstream of the 3′ end of retroposed tRNA sequences. This hypothesis could also be adapted to mRNAs and rRNAs, because some of these functional RNAs in the *N. equitans* genome are encoded on separate genes just like split tRNA [23]. Recently we have reported a novel type of functional tRNA gene in a primitive red alga, *Cyanidioschyzon merolae*, in which the 3′ half of the tRNA sequence lies upstream of the 5′ half in the genome [19]. This ‘permuted’ tRNA might also be considered as a result of the adaptive integration of 5′ and 3′ tRNA halves of ancient eukaryotes or a retroposed product of circular pre-tRNA transcript. Consequently, we have provided a molecular basis for our current understanding of the origin and evolution of tRNAs. Novel tRNA sequences from the upcoming fourth archaeal phylum, the Korarchaeota [37], and other primitive Eukarya and Prokarya separated near the root of the phylogenetic tree will allow further testing of our hypothesis.

## Materials and Methods

### Preparation of tRNA sequences

tRNA sequences were predicted from the genome sequences of the following 45 archaeal species: **Crenarchaeota (15 species):** *Aeropyrum pernix* K1 (Ape), *Caldivirga maquilingensis* IC-167 (Cma), *Cenarchaeum symbiosum* (Csy), *Hyperthermus butylicus* DSM 5456 (Hbu), *Ignicoccus hospitalis* KIN4/I (Ign), *Metallosphaera sedula* DSM 5348 (Mse), *Pyrobaculum aerophilum* str. IM2 (Pae), *Pyrobaculum arsenaticum* DSM 13514 (Par), *Pyrobaculum calidifontis* JCM 11548 (Pca), *Pyrobaculum islandicum* DSM 4184 (Pis), *Staphylothermus marinus* F1 (Sma), *Sulfolobus acidocaldarius* DSM 639 (Sac), *Sulfolobus solfataricus* P2 (Sso), *Sulfolobus tokodaii* str. 7 (Sto), *Thermofilum pendens* Hrk 5 (Tpe). **Euryarchaeota (29 species):** *Archaeoglobus fulgidus* DSM 4304 (Afu), *Candidatus* Methanoregula boonei 6A8 (Cme), *Haloarcula marismortui* ATCC 43049 (HmaI [chromosome I], HmaII [chromosome II]), *Halobacterium* sp. NRC-1 (Hsa), *Haloquadratum walsbyi* DSM 16790 (Hwa), *Methanocaldococcus jannaschii* DSM 2661 (Mja), *Methanococcoides burtonii* DSM 6242 (Mbu), *Methanococcus maripaludis* C5 (MmaC5), *Methanococcus maripaludis* S2 (MmaS2), *Methanococcus vannielii* SB (Mva), *Methanocorpusculum labreanum* Z (Mla), *Methanoculleus marisnigri* JR1 (Mcu), *Methanopyrus kandleri* AV19 (Mka), *Methanosaeta thermophila* PT (Msa), *Methanosarcina acetivorans* C2A (Mac), *Methanosarcina barkeri* str. Fusaro (Mba), *Methanosarcina mazei* Go1 (Mma), *Methanosphaera stadtmanae* DSM 3091 (Mst), *Methanospirillum hungatei* JF-1 (Mhu), *Methanothermobacter thermautotrophicus* str. Delta H (Mth), *Natronomonas pharaonis* DSM 2160 (Nph), *Picrophilus torridus* DSM 9790 (Pto), *Pyrococcus abyssi* GE5 (Pab), *Pyrococcus furiosus* DSM 3638 (Pfu), *Pyrococcus horikoshii* OT3 (Pho), *Thermococcus kodakarensis* KOD1 (Tko), *Thermoplasma acidophilum* DSM 1728 (Tac), *Thermoplasma volcanium* GSS1 (Tvo), uncultured methanogenic archaeon RC-I (Mrc). **Nanoarchaeota (1 species):** *Nanoarchaeum equitans* Kin4-M (Neq).

All genomic information was downloaded from the Microbial Genomes resource of the National Center for Biotechnology Information (NCBI) (http://www.ncbi.nlm.nih.gov/genomes/MICROBES/microbial_taxtree.html), except for the raw sequence data of the *Caldivirga maquilingensis* IC-167 draft genome, which was downloaded from the Department of Energy's Joint Genome Institute (http://genome.jgi-psf.org/draft_microbes/calma/calma.home.html).

Both nonintronic and intronic tRNAs were predicted by 2 tRNA-predicting programs, SPLITS (7, 8) and tRNAscan-SE (17). tRNAscan-SE was run with default parameters for predicting nonintronic tRNAs and intronic tRNAs with canonical introns. SPLITS was run parameters -d 2, -p 0.51, -H 2, and –F 3 for predicting intronic tRNAs with multiple introns. The split tRNA sequences were taken from Randau [30]. All predicted tRNA sequences were manually checked to eliminate false candidates and to correct the intron sequences. All predicted archaeal tRNA sequences are registered in a new tRNA database (Sugahara and Kikuta *et al.*, to be published separately).

### Accession number of Archaeal genomes

[Species (GenBank ID)]: Aeropyrum pernix K1 (BA000002.3), Cenarchaeum symbiosum (DP000238), Hyperthermus butylicus DSM 5456 (CP000493.1), Ignicoccus hospitalis KIN4/I (CP000816.1), Metallosphaera sedula DSM 5348 (CP000682.1), Pyrobaculum aerophilum str. IM2 (AE009441.1), Pyrobaculum arsenaticum DSM 13514 (CP000660.1), Pyrobaculum calidifontis JCM 11548 (CP000561.1), Pyrobaculum islandicum DSM 4184 (CP000504.1), Staphylothermus marinus F1 (CP000575.1), Sulfolobus acidocaldarius DSM 639 (CP000077.1), Sulfolobus solfataricus P2 (AE006641.1), Sulfolobus tokodaii str. 7 (BA000023.2), Thermofilum pendens Hrk 5 (CP000505.1), Archaeoglobus fulgidus DSM 4304 (AE000782.1), Candidatus Methanoregula boonei 6A8 (CP000780.1), Haloarcula marismortui ATCC 43049 (AY596297.1), Halobacterium sp. NRC-1 (AE004437.1), Haloquadratum walsbyi DSM 16790 (AM180088.1), Methanocaldococcus jannaschii DSM 2661 (L77117.1), Methanococcoides burtonii DSM 6242 (CP000300.1), Methanococcus aeolicus Nankai-3 (CP000743.1), Methanococcus maripaludis C5 (CP000609.1), Methanococcus maripaludis C7 (CP000745.1), Methanococcus maripaludis S2 (BX950229.1), Methanococcus vannielii SB (CP000742.1), Methanocorpusculum labreanum Z (CP000559.1), Methanoculleus marisnigri JR1 (CP000562.1), Methanopyrus kandleri AV19 (AE009439.1), Methanosaeta thermophila PT (CP000477.1), Methanosarcina acetivorans C2A (AE010299.1), Methanosarcina barkeri str. Fusaro (CP000099.1), Methanosarcina mazei Go1 (AE008384.1), Methanosphaera stadtmanae DSM 3091 (CP000102.1), Methanospirillum hungatei JF-1 (CP000254.1), Methanothermobacter thermautotrophicus str. Delta H (AE000666.1), Natronomonas pharaonis DSM 2160 (CR936257.1), Picrophilus torridus DSM 9790 (AE017261.1), Pyrococcus abyssi GE5 (AL096836.1), Pyrococcus furiosus DSM 3638 (AE009950.1), Pyrococcus horikoshii OT3 (BA000001.2), Thermococcus kodakarensis KOD1 (AP006878.1), Thermoplasma acidophilum DSM 1728 (AL139299.1), Thermoplasma volcanium GSS1 (BA000011.4), uncultured methanogenic archaeon RC-I (AM114193.2), Nanoarchaeum equitans Kin4-M (AE017199.1).

### tRNA sequence analysis

All mature tRNA sequences (input as tDNA sequences) were aligned using CLUSTALW [38] with the following parameters: Multiple Alignment parameter “Gap Opening” = 22.5; Multiple Alignment parameter “Gap Extension Penalty” = 0.83; Pairwise Alignment parameter “Gap Opening” = 22.5; Multiple Alignment parameter “Delay Divergent Sequence” = 25%. These parameters gave the highest performance in RNA sequence alignment [39]. Aligned tRNA sequences (.aln file) were manually improved by matching the consensus nucleotides conserved among archaeal tRNAs: base 8 (T or C), base 14-15 (AG), base 18-19 (GG), base 21 (A or G), base 33 (T), base 48 (T or C) base 53-58 (GTTC[AG]A), base 60-61 (TC) and base 72 (T or C) [6] to obtain a structural alignment. The neighbor joining tree was constructed using CLUSTALW. Bayesian trees were constructed using MrBayes software v. 3.1.2 [40]. The model of sequence evolution was determined by MrModeltest software v. 2.2. The phylogenetic tree files were visualized using Hypertree [41] and Treeview X software (http://darwin.zoology.gla.ac.uk/rpage/treeviewx/). The mimicry of split tRNA halves were conducted by separating tRNA sequences at positions 34–36 (anti-codon); we defined the 2 separated sequences as 5′ half tRNA (positions 1–33) and 3′ half tRNA (positions 37–73). Sequence similarity was calculated based on the aligned file of mature tRNA sequences (.aln). The anti-codon and CCA sequence was excluded to avoid bias. Networks were visualized with Cytoscape software v. 2.5 [42]. Clustering coefficient *c* is calculated as given equation:
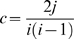
where *i*(*i*-1) refers to the total number of possible connection among *i* nodes, *j* refers to the total number of edge found in the network. Clustering coefficient measures relative linkage among nodes and it is used to understand the topological characteristics of a network (18, 20).

## Supporting Information

Figure S1 (0.03 MB DOC)

Figure S2 (0.18 MB DOC)

Figure S3 (0.76 MB DOC)
