# Supplementary material for: Sequence Evidence in the Archaeal Genomes that tRNAs Emerged Through the Combination of Ancestral Genes as 5′ and 3′ tRNA Halves
Source: PLoS One. 2008 Feb 20;3(2):e1622. doi: 10.1371/journal.pone.0001622 (PMC2237900; doi:10.1371/journal.pone.0001622)
Supplement: Figure S1 — (0.03 MB DOC) [file pone.0001622.s001.doc]

**Supplementary Figure 1.** **Distribution of tRNA clusters in 16 archaeal genera**. The tRNA cluster number (see Figure. 1) is shown for each anti-codon in 16 archaeal genera. Dominant clusters for each anti-codon are colored green. The number of groups for each anti-codon is shown in red.
