# Supplementary material for: Sequence Evidence in the Archaeal Genomes that tRNAs Emerged Through the Combination of Ancestral Genes as 5′ and 3′ tRNA Halves
Source: PLoS One. 2008 Feb 20;3(2):e1622. doi: 10.1371/journal.pone.0001622 (PMC2237900; doi:10.1371/journal.pone.0001622)
Supplement: Figure S2 — (0.18 MB DOC) [file pone.0001622.s002.doc]

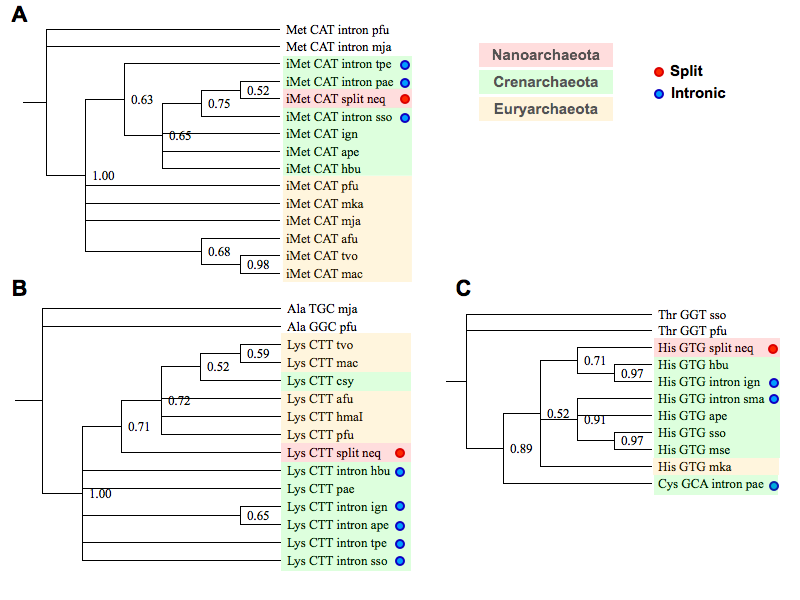


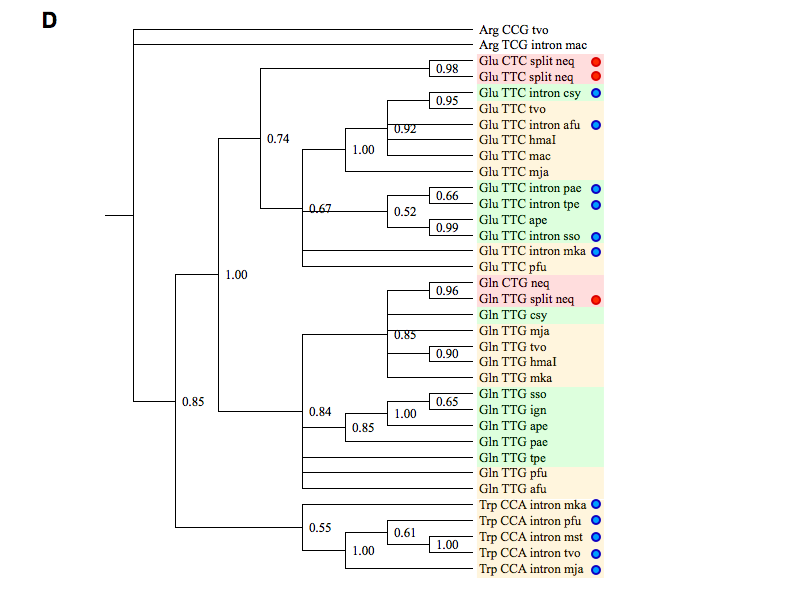


**Supplementary Figure 2. Bayesian phylogenetic trees of tRNA clusters containing split tRNAs.** Five tRNA clusters containing split tRNAs and an adjacent tRNA cluster as an outgroup were compared. (A) Phylogenetic tree of initiator tRNA^iMet^ with tRNA^eMet^ as an outgroup. (B) Phylogenetic tree of tRNA^Lys^ with tRNA^Ala^ as an outgroup. (C) Phylogenetic tree of tRNA^His^ with tRNA^Thr^ as an outgroup. (D) Phylogenetic tree of tRNA^Glu^, tRNA^Gln^ and tRNA^Trp^ with tRNA^Arg^ as an outgroup.
