# Supplementary material for: Sequence Evidence in the Archaeal Genomes that tRNAs Emerged Through the Combination of Ancestral Genes as 5′ and 3′ tRNA Halves
Source: PLoS One. 2008 Feb 20;3(2):e1622. doi: 10.1371/journal.pone.0001622 (PMC2237900; doi:10.1371/journal.pone.0001622)
Supplement: Figure S3 — (0.76 MB DOC) [file pone.0001622.s003.doc]

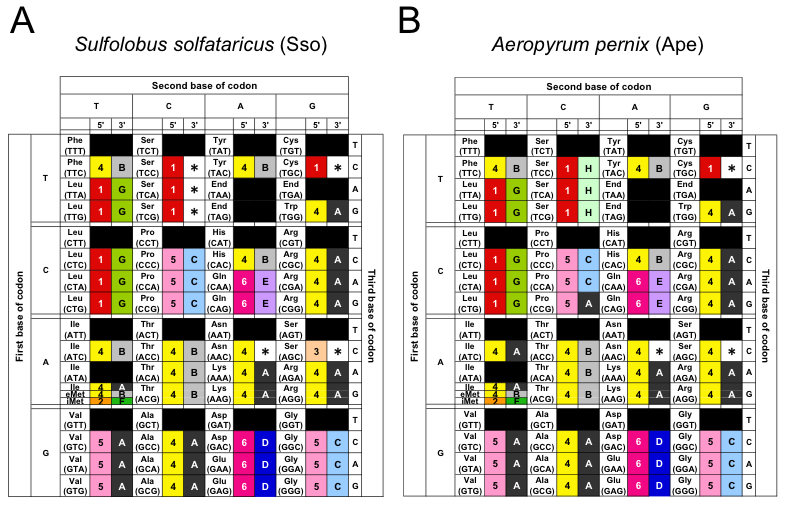


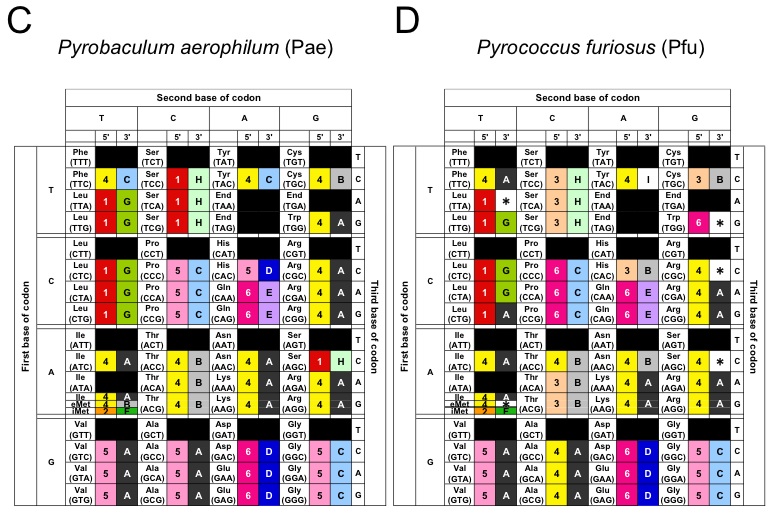


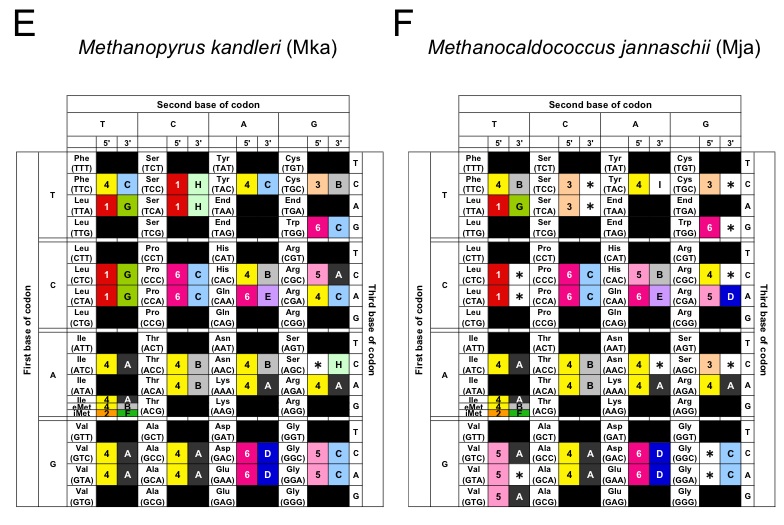


**Supplementary Figure 3. Combinations of 5′and 3′tRNA halves in 6 diverse archaeal species.** The codon table of each species was filled by the group IDs of tRNA halves defined in Figure. 3.
